# Sandwich Structures Reflecting Thermal Radiation Produced by the Human Body

**DOI:** 10.3390/polym13193309

**Published:** 2021-09-28

**Authors:** Jiří Militký, Dana Křemenáková, Mohanapriya Venkataraman, Josef Večerník, Lenka Martínková, Jan Marek

**Affiliations:** 1 Department of Material Engineering, Faculty of Textile Engineering, Technical University of Liberec, 461 17 Liberec, Czech Republic; jiri.militky@tul.cz (J.M.); dana.kremenakova@tul.cz (D.K.); 2 Večerník s.r.o., 468 21 Alšovice, Czech Republic; jvecernik@seznam.cz; 3 Inotex, s.r.o., 544 01 Dvůr Králové nad Labem, Czech Republic; martinkova@inotex.cz (L.M.); marek@inotex.cz (J.M.)

**Keywords:** sandwich structures, far-infrared, functional fabrics, Milife, composite non-woven

## Abstract

Far infrared (FIR) textiles are a new category of functional textiles that have presumptive health and well-being functionality and are closely related to human thermo-physiological comfort. FIR exerts strong rotational and vibrational effects at the molecular level, with the potential to be biologically beneficial. In general, after absorbing either sunlight or heat from the human body, FIR textiles are designed to transform the energy into FIR radiation with a wavelength of 4–14 μm and pass it back to the human body. FIR textiles can meet increased demand for light, warm, comfortable, and healthy clothing. The main aim of this research is to describe the procedure for creating the FIR reflective textile layer as part of multilayer textile structures that have enhanced thermal protection. To develop the active FIR reflecting surface, the deposition of copper nanolayer on lightweight polyester nonwoven structure Milife, which has beneficial properties of low fiber diameters, good shape stability and comfort, was used. This FIR reflective layer was used as an active component of sandwiches composed of the outer layer, insulation layer, active layer, and inner layer. The suitable types of individual layers were based on their morphology, air permeability, spectral characteristics in the infra-red region, and thermal properties. Reflectivity, transmittance, and emissivity were evaluated from IR measurements. Human skin thermal behavior and the prediction of radiation from the human body dependent on ambient conditions and metabolic rate are also mentioned. The FIR reflective textile layer created, as part of multilayer textile structures, was observed to have enhanced thermal protection.

## 1. Introduction

Terms such as “smart” or “intelligent” are used in the context of differentiating new materials and structures from traditional materials and structures [1]. The materials themselves cannot be smart but have only special properties used to construct intelligent structures. Structures that are able to independently evaluate the state of the environment and respond appropriately to it are then referred to as intelligent. Smart textiles are textile structures that are sensitive to external stimuli (various types of radiation, pH, mechanical magnetic or electric fields) and, depending on changes in these stimuli, react reversibly (usually by changing porosity, color [2,3] and shape, among others). Textiles with only multifunctional effects have special properties (e.g., antibacterial activity, ultraviolet protection, electromagnetic shielding, photocatalytic self-cleaning) [4,5], but not reversible reactions. Far infrared (FIR) textiles are typically multifunctional because they have electromagnetic shielding and antimicrobial properties as well. Control of the far-infrared region (further FIR) radiation via the thermal radiation properties of textile layers has a significant effect on localized heating or cooling of the human body under given indoor conditions. Considerable energy savings can be expected by developing radiative heating textiles, as space heating holds a much larger proportion of all the energy consumed in the buildings sector than space cooling.

Clothing for low temperatures below 0 °C, and extremely low temperatures below −15 °C, is necessary to protect humans from dangerous extreme conditions. Fabrics with FIR function can effectively offer thermal retention, may be considered ideal materials for providing warmth retention and can be used for sporting activities such as mountaineering, hiking, as well as working in cold environments [6,7,8]. Known technical solutions for protective clothing against low to extremely low ambient temperatures are based on thick clothing thermal insulation layers, limiting the wearer’s mobility during the activity and increasing the overall weight of the clothing. It is also known that clothing systems composed of thick insulating layers are unable to capture a substantial part of the heat generated by the human body (30% of human heat balance at rest, and more than 30% with physical exertion), which is radiated to the outer layers of clothing and subsequently from the surroundings. To better capture the heat generated by the human body, known thermal insulation garments are sometimes equipped with a layer of aluminum foil to prevent the escape of thermal radiation into the environment. However, these garments have the significant disadvantage that they do not allow the transport of water vapor, which increases the user’s feeling of discomfort and, at low ambient temperatures, moisture accumulates in the garment, which can cause hypothermia. A garment with a layer of perforated aluminum foil is designed to eliminate this disadvantage. However, such perforation is performed mechanically and results in a suit with aluminum foil with relatively large openings, which significantly reduces the effect of such a thermal insulation foil in shielding the thermal radiation produced by the human body. The human body constantly produces heat as a result of basal metabolism, specific bodily activities, and ambient temperature-humidity conditions. The power of the generated heat ranges from about 75 W in sleep to about 1000 W during extreme exertion. Most heat, approximately 85%, is dissipated through the skin. When there is an air gap between the human body and clothing, heat is propagated by skin radiation. Depending on the ambient conditions, this heat is emitted at wavelengths from 4.4 μm with a maximum of 9.4 μm (FIR). It has been verified that this FIR thermal energy has a number of positive effects on the human body. For an indoor environment, where most people are in a sedentary state, more than 50% of the heat generated by the human body is dissipated through infrared radiation. The human body produces FIR, and has severe deficiencies [9,10,11,12]. Existing radiative heating textiles are based on the concept of reflecting human body IR radiation, which is not the most effective IR radiation control approach for localized human body heating. There is a discrepancy between optimal radiative heating performance and good wearability. The decisive influence on the thermal state of a person is heat balance, which expresses the relationship between the amount of heat produced by the organism and the amount of heat removed from the organism to the surroundings. In thermal equilibrium, when there is no heat accumulation in the body, all the heat produced is transferred to the surrounding environment in the form of radiation, flow, conduction, evaporation, and respiration. Based on experiments, it was found that the loss of heat from the body is influenced by the temperature of the surrounding [9,10]. Of the total incident heat energy, 21% is reflected by the surface; 66% penetrates to the corneum, 50% penetrates to the sub-cutaneous tissue, and 99% of the total radiation is absorbed within 3 mm of the surface. For good penetration, the maximum radiation is at 1.2 microns [11,12,13,14,15,16,17]. It is evident that real radiation starts from about 3 μm, where sunlight no longer emits [16]. In the area where the skin emits significantly, it has low reflectance and transmittance [14]. At a wavelength of 2 μm, the skin reflectance is only about 7% and further decreases monotonically above this wavelength. Above wavelengths of 6.5 to 7.5 μm, skin reflectance is zero [14,15,16,17]. Infrared transmittance through the skin, even for the most penetrating beams, is small. About 95% of FIR is absorbed within 2 mm of the skin surface, and 99% within 3 mm [18,19,20]. Particularly in the range of 8–14 μm, FIR is suspected to have many biological effects [21]. This range of wavelengths transfers energy that thermo-receptors in the skin perceive as heat. In previous years, people believed that the optimal wavelength most effective for life is between 8 and 14 μm [22]. Numerous medical studies used FIR with an external heat supply source to demonstrate that FIR wavelengths increase skin microcirculation, improve blood flow of arteriovenous fistulas in hemodialysis patients, extend survival of skin grafts, and have other health-promoting effects. Materials enabling FIR reflection can be used for the creation of so-called heating textiles with the capability of using the heat generated by the human body. These textiles are typically based on highly conductive metallic surface coatings (deposition), with silver or copper being promising candidates. In [23,24], a bilayer nanophotonic structure textile composed of an IR-reflective metallic layer, and an IR-transparent PE layer with embedded nanopores in both layers to simultaneously produce minimal IR emissivity and good breathability, was proposed. In this nanoporous metalized PE design, the embedded nanopores in the metallic layer are smaller than the IR wavelength but larger than water molecules. Multiple layers were also prepared with dual modes of cooling and heating functionalities [25]. In heating mode, it is beneficial to have an inner layer with high reflectivity (low emissivity) in FIR, enabling FIR reflectance, and an outer layer with low reflectivity (high emissivity) in NIR, enabling NIR emission to the human body. In cooling mode, it is necessary to flip the active layers. Apart from FIR transmittance and NIR reflectance of textiles (cooling effects from inside and outside), the surface emissivity of textiles plays an important role. Due to increasing the emissivity of the textile surface, radiation heat flux increases, and the radiative cooling effect is achieved. For obtaining high emissivity levels on the outer surface of textiles, a highly porous layer of carbon material (4–9 μm) has been proposed [25]. A low emissivity surface can be obtained by using a layer of shiny metallic material such as copper (50–150 nm) [25]. These high and low emissivity layers were applied to thick and thin nanoporous polyethylene foil (NanoPE). As a result, two sides of the produced fabric possessed different emissivity levels and different NanoPE thicknesses; therefore, dissimilar heating and cooling effects were achieved. Switching between cooling and heating modes was possible by simple flipping in and out of the fabric. The overall availability of literature for creating textile thermal insulation sandwich structures for shielding of infrared radiation is limited. Our novel research work is a good addition to the knowledge base of the textile material research community.

One promising material for passive radiative heating is copper-coated Milife, due to reflection in the FIR range. Milife is a unique material produced by JX Nippon ANCI Corporation, composed of a machine-directional (MD-oriented) and cross-directional (CD-oriented) layer of PET monofilaments. Both layers are thermally pointed together (see Figure 4). Milifes are supplied in a planar weight of 5 to 60 g∙m^−2^, thicknesses of 0.05 to 0.17 mm, and strength of 20 to 300 N per 5 cm of width. Milife is a promising material for the surface deposition of metals because it is composed of a dense network of monofilaments connected by fixing point spots. PET material can be easily activated for higher surface tension (more than 50 mN·m) by hydrolysis (preferably, in principle) or plasma pretreatment. Plasma pretreatment with autocatalytic activation is commonly used for the metallization of Milife.

This work aimed to create a textile thermal insulation sandwich fabric that can shield infrared radiation in the wavelength range of 4 to 14 µm while maintaining sufficient breathability and eliminating, or at least reducing, the disadvantages of the above technical solutions while increasing the comfort of clothing for users. To create an active FIR reflecting surface, the deposition of copper nanolayer on the lightweight polyester nonwoven structure of Milife was used. This structure is a part of a multilayer sandwich structure for enhanced heat protection. Individual layer geometry, air permeability, and thermal properties were measured. The reflectivity, transmittance, and emissivity were evaluated from IR measurements.

## 2. Materials and Methods

### 2.1. Composition of Sandwiches

**Figure 1:**
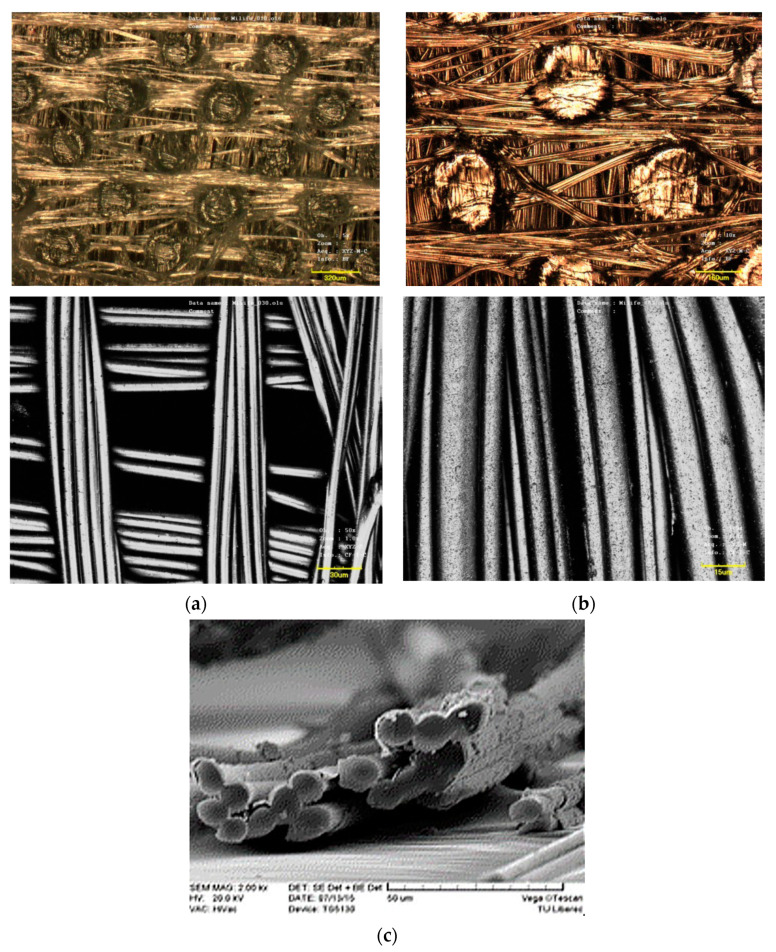
Surface and longitudinal view of Milife at high magnifications: (**a**) original, (**b**) copper coated, (**c**) detail of copper-coated layer.

The aim of this study was to create fabrics with the FIR function based on active FIR reflective textile layer as part of multilayer sandwich textile structures having enhanced thermal protection (see Figure 5). This active FIR reflective textile layer was created by the metallization of a Milife polyester composite nonwoven layer. The copper coating was realized by reduction in a strong alkaline bath containing copper salts at ambient temperature. The copper deposit was about 3 g·m^−2^_,_ dependent on the planar weight of Milife. Copper-coated Milife has enhanced reflectance in the FIR region, and untreated Milife is sufficiently transparent for the NIR range (sun radiation) [26]. The structure of copper-coated Milife is shown in Figure 1.

**Figure 2:**
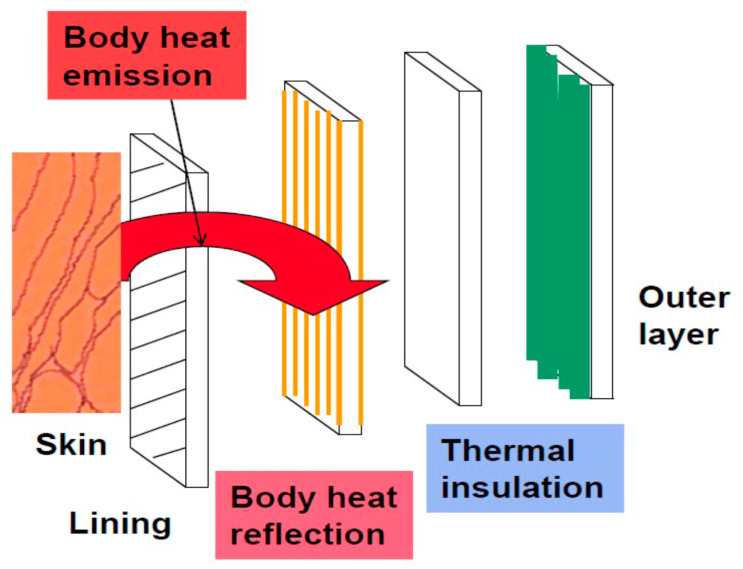
Multilayered sandwich thermal protective system.

The composition of sandwich protective structures from the polyester fibers with different fineness and structural arrangements are shown in Figure 2.

Each layer used for the creation of sandwich structure is characterized in Table 1. The primary aim was to characterize the properties of sandwiches with respect to the order and properties of individual layers. Based on preliminary experiments, the optimal arrangements of individual layers were the outer layer, insulation layer, active layer, and inner layer. The copper-coated Milife fabric (VTM) was provided by surface protective lacquer after coating.

### 2.2. Geometrical Characteristics of Layers 

Real areal weight thickness (under a pressure of 1 kPa) was measured, and layer densities, fiber volume fractions, and volume porosity were calculated by standard formulas. Insulating nonwoven layers were made with high a value of volume porosity of 99% (some are shown in Table 2). Copper-coated Milife has a volume porosity of 78%. The volume porosity of the upper fabric is about 50%, and for the lining samples, 89%. The thickness of the sandwiches is the sum of the thicknesses of the individual layers.

### 2.3. Air Permeability

Air permeability was measured on an FX 3300 instrument (Air Permeability Tester III, Schwerzenbach, Switzerland) according to ČSN EN ISO 9237. The measured area of the samples was 25 cm^2^. Ten measurements were performed on each sample. Arithmetic averages of air permeability values and lower and upper limits of 95% confidence intervals of the population mean air permeability values were calculated. The air permeability of selected layers and sandwiches was measured in the direction from the lining to the top layer, and vice versa.

### 2.4. Measurement of Thermal Properties

The thickness measurement, thermal conductivity, and thermal resistance were performed on an Alambeta instrument according to internal standard No. 23-304-02/01. Said quantities were measured on the principle of heat conduction. The measurement was performed at normally set parameters of the device with pressure P = 200 Pa, at air temperature in the laboratory t = 22.8–23.2 °C, and relative air humidity φ = 39%. Each measurement was performed five to ten times. Average values and 95% confidence intervals of the population mean values of thickness, thermal conductivity in W m^−2^·K^−1^, and thermal resistance in m^2^ K·W^−1^, were calculated. The thermal resistance in clo units was calculated. The thermal properties of individual layers and selected sandwiches were measured. For very thin fabrics (thickness less than 0.5 mm) of the Milife inner layer (lining) and the top layer (fabric TKU3), it was not possible to measure these quantities directly. Therefore, they were layered on top of each other.

### 2.5. Measurement of the Degree of Thermal Insulation

Measurement of the degree of thermal insulation is based on the measurement of temperature. It follows from the measuring principle that, in this case, the heat is not propagated by conduction but by radiation. The surface temperature of the thermostat was chosen to be 40 °C. Fabric from a viscose/cotton/polyester mixture was chosen as the base layer (3 mm away from the thermostat plate), which did not affect the radiant heat transfer. The temperature of the base layer was expressed as *T*_p_ (°C). Measured samples were attached to this base layer in the frame. Their surface temperature *T*_f_ (°C) was measured by an infrared radiation detector (thermal camera, pyrometer) after 1 min of stabilization. The distance of the detector from the frame was 40 cm. The room temperature *T*_a_ (°C) was also measured. The relationship for computing the thermal insulation index *I*_r_ (−) of thermal radiation is:

(1) $$I_{r}=\frac{T_{p}\;-{\;T}_{f}}{T_{p}\;-{\;T}_{a}}$$

For fabrics that completely transmit radiant heat radiation, *I*_r_ = 0, since the temperature of the base layer *T*_p_ is the same as the surface temperature of the fabric *T*_f_ (°C). For completely thermally insulating materials, *I*_r_ = 1, and, in this case, the fabric’s surface temperature corresponds to the ambient temperature *T*_a_. 

When measuring the surface temperature with an IR detector, the emissivity must be set. It was assumed that most textiles have an emissivity close to one, and a value of 0.95 was set. A problem arose when measuring the surface temperature of metalized Milife fabrics, which have lower emissivity values. If for a certain value of emissivity *ε_Z_* (−) the temperature *T_Z_* (°C). is detected, and the real surface temperature of the object is *T_F_* (°C), it the real emissivity *ε_F_* (−) is calculated according from Stefan-Boltzmann’s law:

(2) $$\varepsilon_{F}=\varepsilon_{Z}{\left(\frac{T_{Z}}{T_{F}}\right)}^{4}$$

A black body with high thermal conductivity and high emissivity was placed on the part of the fabric surface (e.g., copper foil painted with black nonreflective coating, *ε_Z_* = 0.98). The thermostat was set to a constant temperature. The detector measured the surface temperature of the black body *T*_Z_ and the surface temperature of the fabric *T*_F_ at the set emissivity *ε_Z_*. The emissivity of the fabric *ε_F_* was then calculated according to Equation (2). In our case, a sticker with the specified emissivity *ε_Z_* = 0.96 was used as the black body. The surface of the label and the sample were heated with a metal plate for 20 s. The emissivity was calculated using FLIR Research IR Max software. 

### 2.6. Measurement of Reflectance, Transmittance, and Calculation of Absorbance

Reflectance and transmittance were measured on a Mid-IR IntegratIR^®^ from PIKE technologies^®^, Fitchburg, WI, USA (integration sphere). The device is designed to measure the diffusion reflectance of materials, and is a modular extension of an infrared spectroscope (FTIR method). The principle of the test is to measure the sample for the reflection of infrared rays with a wavelength range from 2 to 20 μm. The spectral absorbance *A_λ_* (−) was also calculated according to the relationship:

*A_λ_* = 1 − *R_λ_* − *T_λ_* (3)

where *R_λ_* (−) is the spectral reflectance and *T_λ_* (−) is the spectral transmittance at the wavelength *λ* (µm).

## 3. Results and Discussion

### 3.1. Geometry and Air Permeability

Measured and calculated geometrical characteristics of individual layers are shown in Table 2. The relationship between areal weight *G* (g·m^−2^) and thickness *h* (mm), within the same structure, represented by constant volume porosity *P_o_* (−), (*ρ* = 1360 kg·m^−3^ was the density of the polymer) is given by:

*G* = (1 − *P_o_*) *h·ρ* (4)

Therefore, the areal weight is directly proportional to the thickness. For these reasons, the characteristic parameter of the layers is their thickness.

Air permeability (mm/s) of individual layers are shown in Table 3 and selected sandwiches are shown in Table 4.

In the following, we use abbreviations LL as the lower limit of 95% confidence interval, and UL as the upper limit of 95% confidence interval.

The relationship between air permeability and relative pressure drop (ratio of pressure drop Δ*p* and fabric thickness *h*) can be expressed according to Darcy’s linear relationship [27] or the Ergun model [28]. The Ergun model is based on the flow of fluids through a porous stuffed layer according to the relationship:

(5) $$\frac{\Delta p}{h}=\frac{\upsilon }{K_{1}}v_{f}+\frac{\rho_{a}}{K_{2}}v_{{}_{f}}^{2}$$

where Δ*p* (Pa) is the pressure drop, *h* (m) is the thickness of the fabric, υ (kg·m^−1^s^−1^) is the viscosity of the air, *ρ*_a_ (kg·m^−3^) is the density of the air, and *v_f_* (m·s^−1^) is the velocity of the airflow. *K*_1_ and *K*_2_ are the so-called viscous and inertia factors defined by the relationships:

$$K_{1}=\frac{\varepsilon^{3}d^{2}}{150{\left(1-\varepsilon \right)}^{2}}\;K_{2}=\frac{\varepsilon^{3}d}{1.75(1-\varepsilon )}$$

where *ε* (−) is the volume porosity and *d* (m) is the characteristic length (here fiber diameter). The relative pressure drop is a quadratic function of the air permeability, and the air permeability corresponds to the airflow velocity *v_f_*. In the Ergun model (5), the first term corresponds to Darcy’s linear relation. Using assumption of the same material, constant volume porosity, and a constant pressure drop, the air permeability *P*_r_ (mm/s) depends only on the thickness of the fabric, and Equation (5) can be written in the form:

(6) $$\frac{\Delta p}{h}=a_{1}P_{r}+a_{2}P_{r}^{2}$$

where coefficients a_1_ and a_2_ have units corresponding to the requirement of Equation (6) dimensional homogeneity.

**Figure 3:**
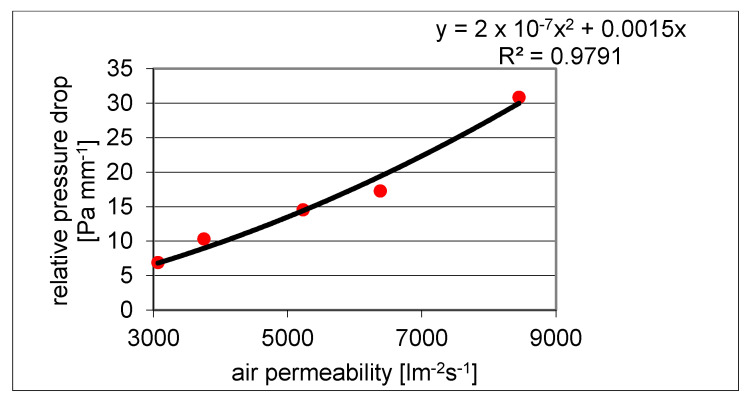
Influence of relative pressure drop on air permeability.

Using linear least squares regression, the parameters a_1_ and a_2_ of a 2nd degree polynomial without an absolute term (corresponding to Equation (6)) were estimated. In Figure 3, the dependence of the air permeability *P*_r_ (here, the independent variable) and the relative pressure drop in the layers is shown (highest point corresponds to an insulation layer with areal density 100 g/m^2^).

It was found that a lower level of bulk porosity led to lower air permeability. The lowest volume porosity of about 50% had a Ripstop weave fabric designed for the top layer, which also had the lowest air permeability. Nonwoven fabrics have a high constant porosity of about 99%, but an increase in thickness causes a decrease in air permeability. The copper plating of nonwoven Milife should lead to a reduction in porosity and air permeability. In some cases, this is not true, which may be due to variability in the porosity of the original gray fabric or deformation during continuous production. The use of a protective lacquer against corrosion and wear on the surface of the Milife fabric did not statistically significantly reduce air permeability. The air permeability of sandwiches depended on the order of the layers. If the first layer was the least porous layer, the air permeability of the sandwich was statistically significantly lower than when the first layer was a lining with high porosity. Air permeability of sandwiches was in a range suitable for clothing purposes [11,29], and the presence of Milife copper-coated layer was not critical.

### 3.2. Thermal Characteristics 

The results of thermal characteristics measurements are given in Table 5. The same characteristics for multiple layers of thin materials and sandwiches are shown in Table 6. 

**Figure 4:**
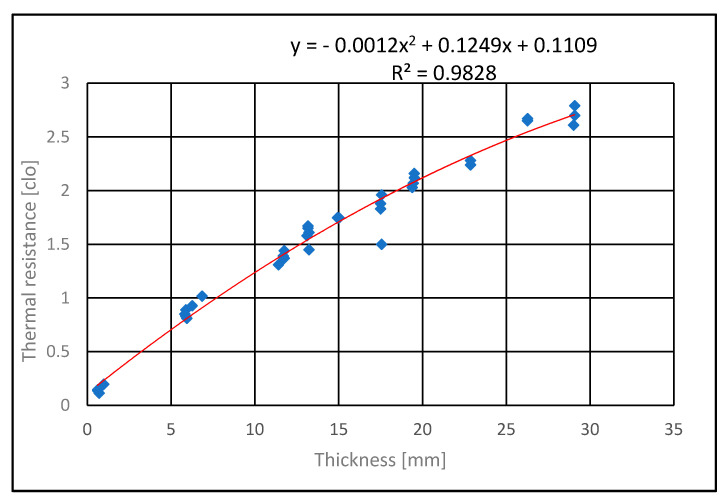
Influence of layer/sandwich thickness on thermal resistance (the more samples of insulation layer are included).

**Figure 5:**
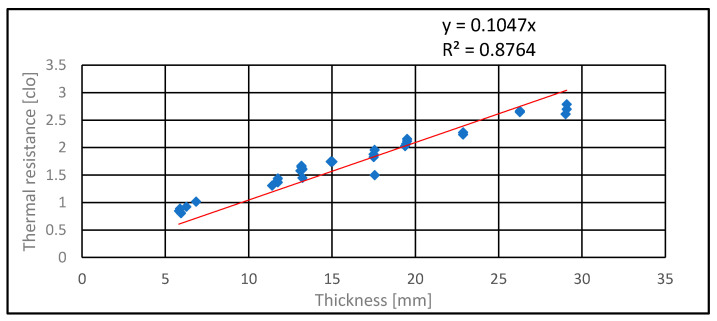
Thermal resistance at a constant thermal conductivity of layers (the more samples of insulation layer are included).

The resulting thermal resistance values in clo units for individual layers and all types of sandwiches are plotted against the thickness [mm] in Figure 4 and Figure 5. In Figure 4, all data are fitted by a polynomial of the 2nd degree the using linear regression:


R_c_ = a_0_ + a_1_ h + a_2_ h^2^ (7)

The parameters found were a_0_ = 0.1129, a_1_ = 0.1268, a_2_ = −0.0013. Their units correspond to the requirement of Equation (7) dimensional homogeneity. The quadratic regression model is shown in Figure 4.

In Figure 5, thermal resistance in clo units is selected except for thin layered textiles (Table 6). The model R_c_ = a h is used. The slope of the regression line $a=\frac{10^{-3}}{0.155\;\lambda }$ is related to the thermal conductivity of individual layers. This corresponds to the assumption that all layers have nearly the same thermal conductivity (a = 0.1047 clo/mm, λ = 0.062 W∙m^−1^∙K^−1^).

The thermal resistance of the layers measured on the Alambeta was not affected by their order. By layering thin conductive layers, there was no significant increase in thermal resistance by conduction. The total thermal resistance by conduction for a thin layer of copper-coated Milife and a layer of insulating nonwoven fabric was approximately equal to the thermal resistance of the insulating fabric. The results show that the thermal resistance of the layers depends mainly on their thickness. Using a polynomial of the 2nd degree, the thermal resistance and thermal conductivity of thin layers can be calculated at the limit of the thermal conductivity of air (0.024 W∙m^−1^∙K^−1^). This corresponds to experimental results from the Alambeta instrument. The thermal resistance changes can therefore be tuned by insulating nonwoven layer porosity and thickness. The thermal resistance of individual layers were similar to values obtained in previous work [29].

### 3.3. Degree of Resistance against Radiation

Thermal insulation index against radiation *I*_r_ of individual layers of textiles is given in Table 7.

To calculate the thermal insulation index against the radiation *I*_r_ (see Table 7) of copper-coated Milife, the surface temperatures were recalculated according to the real emissivity values. The results were mean *I*_r_ = 0.32, LL = 0.25, and UL = 0.40. The result of emissivity measurements of metallized Milife textile were mean of *ε* = 0.487 LL = 0.449 and UL = 0.524. For comparison, the emissivity values of polyester (foil) are in the range 0.75–0.85, copper 0.4–0.8, and nickel 0.2–0.5. The thermal insulation index *I*_r_ of variously combined sandwiches is shown in the Table 8. For comparison, an active layer with an aluminum coating (2Al) was also used.

It was found that the emissivity values of gray polyester nonwoven materials were in the range usual for polyester materials, but the emissivity of textiles is higher than about 0.95 [12,29]. The emissivity of copper-coated Milife nonwoven materials was statistically significantly lower, and decreased to 0.49. The thermal insulation index was statistically significantly higher for copper-coated layers. The insulation index of sandwiches increased with thickness and was also affected by composition. The insulation index of sandwiches was statistically significantly higher than the insulation layers themselves (FT 200). The best results were achieved by sandwiches that contained a laminate of the inner layer PL07, copper-coated Milife layer, an insulating layer (FT 200), and the upper layer TKU3. When the heat flux changed from the top layer through the insulation fabric to the copper-coated Milife layer, there was a statistically significant reduction in the insulation index. When removing the copper-coated Milife layer from the sandwich, there was a statistically significant decrease in the thermal insulation index, which was still at a high level. The thermal insulation index of this sandwich corresponded to a sandwich where the copper-coated Milife layer was replaced by a TKU3 fabric with an aluminum coating (2Al). When changing the direction of heat flow in these sandwiches, the differences in the insulation index were statistically insignificant. This is in accordance with our previous results [29].

### 3.4. Infrared Radiation

The reflectance, transmittance, and absorbance at a wavelength of 10 µm for the individual layers and sandwiches are shown in Table 9 and Table 10. This wavelength represents the maximum emission value of the human body.

It was found that the reflectance of electromagnetic radiation in the range of 2 to 20 µm was significantly affected by the extent of the copper-coated layer. The highest reflectance of 80% was measured for the copper-coated Milife layer with an areal weight of 30 g∙m^−2^. Nonwoven FT200 acrylate bonded insulating fabrics have a very low level of reflectance and transmittance, and thus a high level of absorbance greater than 90%. When incorporating copper-coated, active, reflective layers into sandwiches, the reflectance is reduced. The use of a fabric with a higher reflectance in the sandwich leads to a higher reflectance of the sandwich in the same order of layers. The sandwich PL07 + VTM + FT200 + TKU3 reduced the reflectance to about 20. The reflectance of PL07 + FT200 + TKU3 sandwiches was reduced to 10%. The enhanced reflectance in the FIR region is the main benefit of the presence of a copper-coated active layer. Similar results were presented and are available in [25].

## 4. Conclusions

The preparation of FIR functional fabrics requires the proper selection of surface modification or particulate systems as fillers for obtaining the reflection of FIR generated by the human body. Benefit is derived by using materials with low fiber diameters, good shape stability, and comfort-related properties. Our research revealed that the composite nonwoven fabric Milife with a surface covering of a particulate-based copper layer is a good FIR reflective textile layer. It was crucial to maintain the order of the reflective layer in the sandwich and the reflective fabric should be placed as close as possible to the source of electromagnetic radiation. Nonwoven acrylate bonded fabrics demonstrated very good thermal insulating behavior, and polyester canvas, and Ripstop were good candidates for the outer layer of sandwich structures. Using this sandwich, the heat produced by the human body could be used to improve thermal insulation. The FIR reflective textile layer thus created, as part of multilayer textile structures, was observed to have enhanced thermal protection. These fabrics can have a significant effect on localized heating or cooling of the human body under given indoor conditions while meeting the demands for light, warm, comfortable, and healthy clothing

## Figures and Tables

**Table 1:** Individual layers.

Figure	Material	Composition	Function	Areal Weight Nominal (g∙m^−2^)
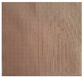	TK U3	Polyester canvas, Ripstop	Outer layer	74
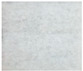	FT 200	Polyester nonwoven, acrylic binder	Insulation layer	200
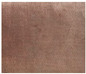	VTM	Milife copper-coated polyester nonwoven	Active layer	30
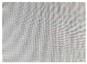	PL 07	Polyester knitted, filet	Lining	67

**Table 2:** Geometrical characteristics of layers.

Layer	Thickness (mm)	Areal Weight Real (g∙m^−2^)	Density (kg∙m^−3^)	Volume Portion (-)	Volume Porosity (-)
TK U3	0.117	75.22	642.91	0.473	0.527
VTM	0.102	35.600	349.020	0.257	0.743
PL 07	0.2435	68.160	279.918	0.206	0.794
FT 200	13.750	209.80	15.26	0.011	0.989

**Table 3:** Air permeability of individual layers.

Layer	Air Permeability (mm/s)	LL (mm/s)	UL (mm/s)
TK U3	71.3	70.2	72.3
VTM	1174	1083	1265
FT 200	5229	5172	5286
PL 07	7860	7707.4	8012.6

**Table 4:** Air permeability of sandwiches.

Sandwich Type	Air Permeability (mm/s)	LL (mm/s)	UL (mm/s)
PL 07/FT 200	3842	3774	3910
FT 200/PL 07	3780	3653	3907
PL 07/VTM	1124	1063	1185
PL 07/FT 200/VTM	992	936	1048
PL 07/FT200/VTM/TKU3	152	150	153

**Table 5:** Thermal conductivity and thermal resistance of insulating layer (heat conduction).

Layer	Thermal Conductivity λ (W m^−1^ K^−1^)			Thermal Resistance R_c_ (m^2^ K W^−1^)			Thermal Resistance (clo)
	Mean	LL	UL	Mean	LL	UL	
FT 200	0.0565	0.0559	0.0572	0.245	0.241	0.250	1.58

**Table 6:** Thermal conductivity and thermal resistance of thin multiple layers and sandwich.

Material	Thickness (mm)	Thermal Conductivity λ (W m^−1^ K^−1^)			Thermal Resistance R_c_ (m^2^ K W^−1^)			Thermal Resistance (clo)
		Mean	LL	UL	Mean	LL	UL	
VTM 6 layers	0.612	0.0363	0.0351	0.0375	0.0168	0.0167	0.0169	0.1458
PL07 4 layers	0.974	0.0429	0.0424	0.0435	0.0226	0.0222	0.0229	0.1963
TKU3 6 layers	0.704	0.0535	0.0518	0.0552	0.0132	0.0131	0.0132	0.1144
TKU3/FT200/VTM/PL07	15.017	0.0552	0.0541	0.0564	0.2706	0.2645	0.2767	1.75

**Table 7:** Thermal insulation index of insulating, inner and upper layers.

Layer Type	Index *I*_r_ (-)	LL (-)	UL (-)
TKU3	0.32	0.31	0.33
FT200	0.52	0.49	0.56
PL07	0.16	0.15	0.18

**Table 8:** Thermal insulation index of different sandwiches, including one with alumina coating (direction from the lining to the next layers/vice versa).

Sample Type	Thickness (mm)	Index *I*_r_ (-)	LL (-)	UL (-)
PL07 + FT200 + TKU3	13.473	0.87/0.92 *	-	-
PL07 + VTM + TKU3	0.476	0.36/0.45 *	-	-
PL07 + VTM + FT200 + TKU3	13.576	0.95/0.78	0.92/0.77	0.97/0.79
TKU3 2Al + FT200 + TKU3	13.364	0.83/0.84	0.80/0.83	0.85/0.86

* only one measurement.

**Table 9:** Reflectance, transmittance, and absorbance of individual layers at a wavelength of 10 µm.

Layer	Reflectance (%)			Transmittance (%)			Absorbance (%)
	Mean	LL (%)	UL (%)	Mean	LL (%)	UL (%)	
VTM	81.78	80.95	82.61	15.27	12.14	18.39	2.5
PL07	6.14	5.88	6.41	34.54	33.64	35.43	59.32
TKU3	8.95	8.90	9.00	9.92	9.57	10.27	81.13
FT200	3.81	2.64	4.98	2.12	1.55	2.68	94.07

**Table 10:** The reflectance of selected sandwiches at a wavelength of 10 µm.

Sandwich Type	Reflectance (%)		
	Mean	LL	UL
PL07 + FT200 + TKU3	9.790	9.374	10.207
PL07 + VTM + FT200 + TKU3	21.167	-	-

## Data Availability

The data presented in this study are available on request from the corresponding author.
